# Predictors of Postoperative Pain in Pediatric Patients Undergoing the Nuss Procedure for Pectus Excavatum

**DOI:** 10.1111/ases.70395

**Published:** 2026-11-08

**Authors:** Sayaka Matsumoto, Koichi Deguchi, Naoko Uga, Tasuku Kodama, Kengo Nakahata, Motonari Nomura, Takehisa Ueno, Miho Watanabe, Hiroomi Okuyama

**Affiliations:** ^1^ Department of Pediatric Surgery The University of Osaka Osaka Japan; ^2^ Department of Gastrointestinal and Pediatric Surgery Tottori University Tottori Japan

**Keywords:** Nuss procedure, pectus excavatum, postoperative pain

## Abstract

**Purpose:**

The purpose of this study was to identify the primary predictors of postoperative pain in pediatric patients undergoing the Nuss procedure, with the aim of enhancing postoperative pain management.

**Methods:**

This retrospective study analyzed data from 40 pediatric patients who had undergone the Nuss procedure for pectus excavatum. Patient demographics, postoperative outcomes, postoperative numerical rating scale (NRS) scores, and the administration of analgesic medications were analyzed statistically.

**Results:**

Age at the time of surgery was identified as the only independent predictor of postoperative pain severity on postoperative day (POD) 5 in the multivariable analysis (*p* = 0.036). No significant differences were observed in other postoperative outcomes. Pain severity, measured by the NRS on POD 1 and POD 5, did not significantly differ between female and male patients. However, older patients reported significantly higher NRS scores compared to younger patients on both POD 1 (*p* = 0.022) and POD 5 (*p* = 0.018). The administration of analgesic medications did not significantly differ by age or sex.

**Conclusion:**

Older pediatric patients experienced more severe postoperative pain than their younger counterparts following the Nuss procedure.

## 1 Introduction

Pectus excavatum is one of the most prevalent congenital thoracic deformities [1]. The Nuss procedure, which involves the insertion of a metal bar through small incisions to correct this deformity, presents a simpler and less invasive alternative to traditional thoracotomy [2]. Despite its minimally invasive nature, pediatric patients undergoing the Nuss procedure frequently experience severe and sometimes intolerable postoperative pain [3]. This pain is not only distressing but is also associated with postoperative complications, such as atelectasis due to impaired sputum clearance [4, 5], which can lead to extended hospital stays. Therefore, optimizing postoperative pain management is essential for facilitating recovery and minimizing the risk of complications.

Given the complexity and subjectivity of pain, coupled with significant interindividual variability in pain perception [6], a thorough evaluation of postoperative pain is imperative. Identifying risk factors for severe postoperative pain may aid in the development of more effective pain management strategies. Although prior studies have reported inconsistent findings regarding postoperative pain in pediatric populations, factors such as female sex, preoperative anxiety, and the type of surgical procedure have been identified as potential predictors of postoperative pain in other surgical contexts [7, 8]. Nevertheless, despite the notable severity of postoperative pain associated with the Nuss procedure, few studies have specifically examined predictors of postoperative pain following this intervention.

Therefore, the purpose of this study was to identify significant predictors of postoperative pain after the Nuss procedure, with a particular emphasis on age and sex.

## 2 Methods

This retrospective study included patients aged < 20 years who had undergone the Nuss procedure for pectus excavatum at Osaka University Hospital between January 1, 2016, and December 31, 2023. Patients who required reoperation during the same hospital admission were excluded.

Preoperative assessments encompassed symptoms, physical examinations, chest imaging, and pulmonary function tests. The severity of the pectus defect was assessed using the Haller index, which was calculated from preoperative chest computed tomography (CT) images as the ratio of the maximum transverse internal diameter of the chest to the minimum anteroposterior distance between the posterior surface of the sternum and the anterior surface of the vertebral body [9]. Patients were deemed candidates for surgical intervention if they exhibited cardiopulmonary physiologic impairment attributable to the thoracic deformity or experienced significant negative psychosocial effects. In cases of asymmetrical pectus excavatum, the angle of sternal rotation was evaluated using CT imaging, and costal cartilage resection was indicated in selected severe cases. Briefly, the three most depressed costal cartilages were partially resected through a mini‐thoracotomy, with the implantation of one or two metal bars [10].

Postoperative pain management adhered to a standardized institutional protocol that included a combination of thoracic epidural analgesia and intravenous analgesics, such as nonsteroidal anti‐inflammatory drugs (NSAIDs), acetaminophen, and fentanyl. All patients underwent thoracic epidural catheter placement and received continuous epidural infusion postoperatively as the primary modality for pain control. Epidural catheter tip position was routinely confirmed by radiography. Continuous epidural infusion of 0.2% ropivacaine was initiated at a weight‐based dose according to the institutional anesthesiology protocol and titrated according to analgesic requirements. The protocol also allowed patient‐controlled additional bolus doses. In cases where epidural analgesia proved insufficient, secondary pain control measures included scheduled acetaminophen, on‐demand NSAIDs, and, when necessary, intravenous fentanyl patient‐controlled analgesia (IV‐PCA).

Data regarding patient demographics, disease characteristics, surgical procedures, postoperative outcomes, postoperative numerical rating scale (NRS; 0 indicating “no pain” and 10 indicating “worst pain imaginable”) scores, as well as the type and frequency of analgesic use, were extracted from electronic medical records. NRS scores were evaluated once each morning by nursing staff throughout the postoperative period. The frequency of additional analgesic medication use was defined as the total number of extra pain control interventions, including NSAIDs, acetaminophen, and fentanyl, regardless of the route of administration. To evaluate the impact of age, patients were categorized into a younger group (age ≤ 10 years) and an older group (age > 10 years). The cutoff of 10 years was selected to approximate the onset of pubertal growth while maintaining a balanced distribution of patients between the two groups.

All statistical analyses were conducted using EZR (version 1.63) [11], a graphical user interface for R, and figures were created using GraphPad Prism (version 10.6.1). Fisher's exact test was used for categorical variables, and Mann–Whitney *U* test was used for nonparametric continuous variables to compare postoperative pain‐related factors between groups. Both univariable and multivariable regression analyses were performed to identify predictors of postoperative pain severity. Due to the limited sample size, the number of covariates was restricted. The multivariable model included sex, age, Haller index, costal cartilage excision, and the number of bars inserted as clinically relevant potential confounders. All statistical tests were two‐sided, and *p* < 0.05 were considered statistically significant.

## 3 Results

Between January 1, 2016, and December 31, 2023, a total of 43 patients underwent the Nuss procedure for pectus excavatum. Three patients who required reoperation during the same hospital admission were excluded, resulting in a final cohort of 13 female patients and 27 male patients for analysis. The median age of the patients was 10 years (IQR, 8–13 years). The younger and older groups comprised 25 and 15 patients, respectively.

The patient demographics are summarized in Table 1. As expected, older patients were significantly taller (*p* < 0.001) and heavier (*p* < 0.001) than their younger counterparts, and male patients were taller than female patients (*p* = 0.045). No other significant differences in baseline characteristics were observed between the age or sex groups. Eight patients (20%) had comorbidities: bronchial asthma (*n* = 3), a history of congenital diaphragmatic hernia repair (*n* = 2; one patient also had a ventricular septal defect), attention‐deficit/hyperactivity disorder (*n* = 1), a history of surgery for bronchial atresia (*n* = 1), and a history of congenital pulmonary airway malformation repair (*n* = 1). Mean NRS scores and the frequency of additional analgesic use from postoperative day (POD) 0 to POD 5, stratified by sex and age, are shown in Figure 1 and Figure 2, respectively. Older patients consistently reported more severe pain (NRS > 5), and the frequency of additional analgesic use remained elevated through POD 5 (Figure 2).

**TABLE 1:** Patient demographic and surgical characteristics, stratified by age and sex.

	Overall	Younger (age ≤ 10 y)	Older (age > 10 y)	*p*	Female	Male	*p*
	(*n* = 40)	(*n* = 25)	(*n* = 15)		(*n* = 13)	(*n* = 27)	
Age (years)	10 (8–13)	9 (8–10)	13 (12.5–14)	< 0.001*	9 (8–13)	10 (8.5–12.5)	0.60
Height (cm)	140.5 (131.2–157.5)	133.4 (126.5–140.0)	162.6 (152.7–165.5)	< 0.001*	135.2 (124.0–150.0)	147.0 (133.5–163.6)	0.045*
Weight (kg)	28.5 (25.6–44.8)	26.0 (24.1–28.2)	44.6 (35.0–50.0)	< 0.001*	27.5 (24.5–34.3)	31.6 (26.3–47.7)	0.10
Haller index	4.58 (4.03–6.04)	4.76 (4.20–6.50)	4.31 (3.93–5.20)	0.30	4.20 (3.98–5.12)	4.92 (4.29–7.11)	0.10
Comorbidity	8 (20%)	5 (20%)	3 (20%)	1.00	5 (38%)	3 (11%)	0.09
Surgery time (min)	111.5 (81.0–169.8)	95.0 (76.0–160.0)	122.0 (99.5–187.0)	0.09	127.0 (83.0–199.0)	105.0 (79.0–155.5)	0.33
Number of bars	1 (1–1)	1 (1–1)	1 (1–2)	0.22	1 (1–1)	1 (1–1)	0.97
1 bar (*n*)	31 (78%)	21 (84%)	10 (67%)		10 (77%)	21 (78%)	
2 bars (*n*)	9 (23%)	4 (16%)	5 (33%)		3 (23%)	6 (22%)	
Costal cartilage excision	11 (28%)	6 (24%)	5 (33%)	0.72	5 (38%)	6 (22%)	0.45

*Note:* Continuous variables are presented as the median (interquartile range [IQR]), and categorical variables as *n* (%).

* *p* < 0.05 indicates statistical significance.

**FIGURE 1:**
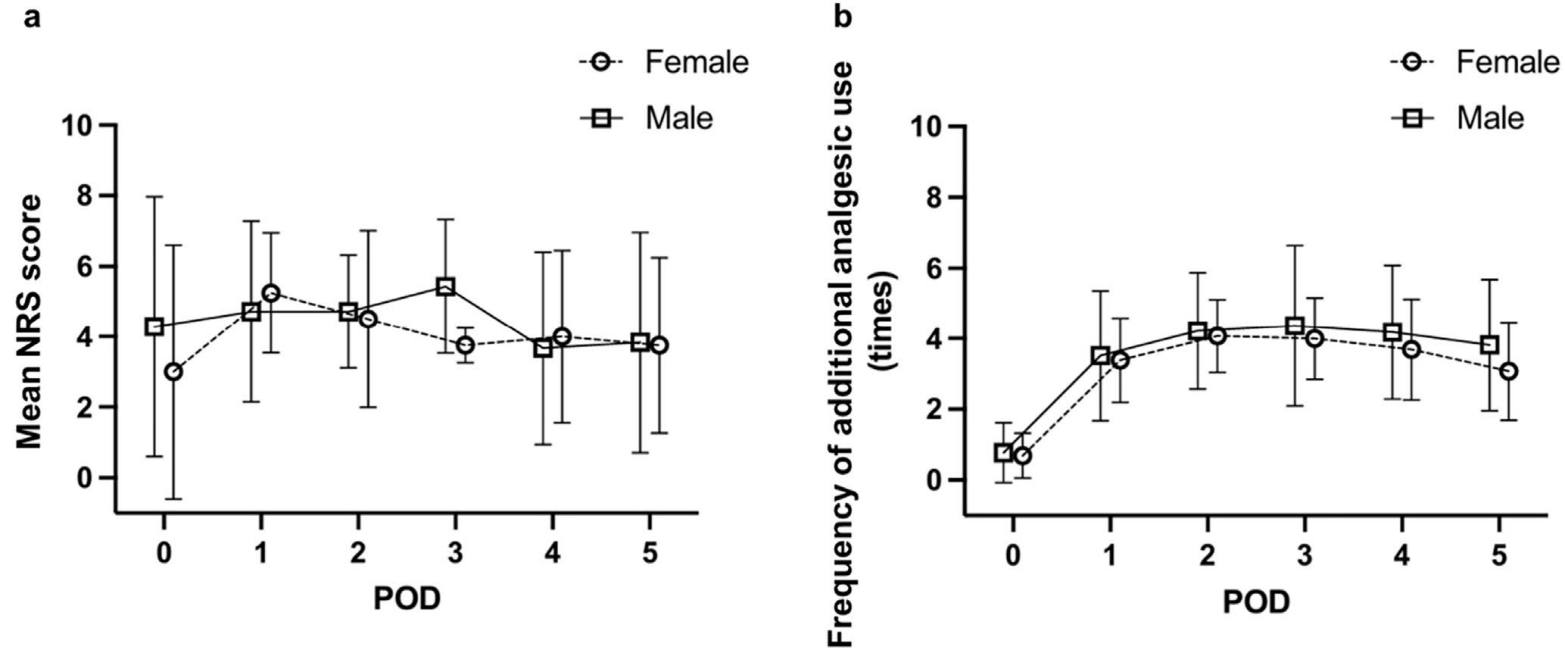
(a) Mean NRS scores and (b) the frequency of additional analgesic use from POD 0 to POD 5, stratified by sex. NRS, numerical rating scale; POD, postoperative day. Data are presented as mean ± SD.

**FIGURE 2:**
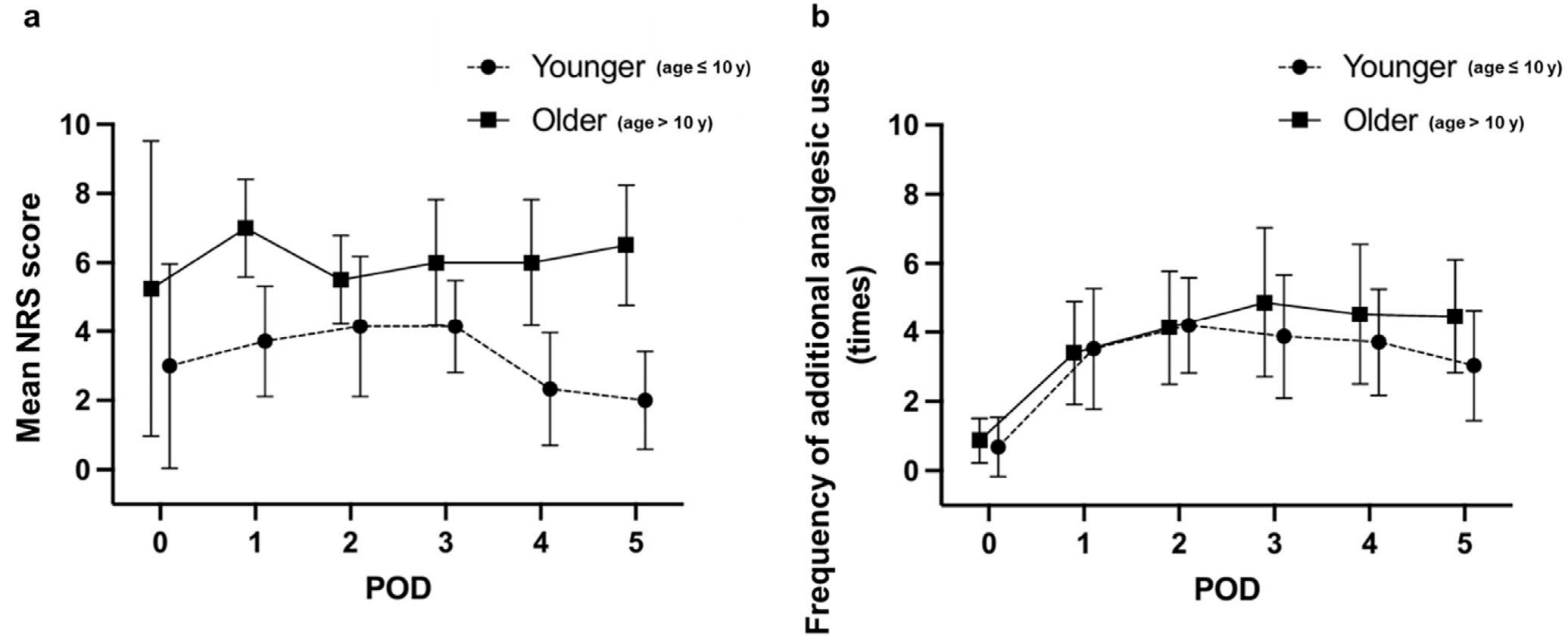
(a) Mean NRS scores and (b) the frequency of additional analgesic use from POD 0 to POD 5, stratified by age. Data are presented as mean ± SD.

Univariable regression analyses were first performed to explore associations between candidate predictors and postoperative pain severity on POD 5. Subsequently, a multivariable regression analysis including sex, age, Haller index, costal cartilage excision, and the number of bars inserted was conducted to adjust for potential confounding factors. Age remained the only significant independent predictor of postoperative pain severity on POD 5 in the multivariable analysis (*p* = 0.036) (Table 2).

**TABLE 2:** Univariable and multivariable regression analyses of predictors of postoperative pain severity on postoperative day 5.

Predictor	Univariable analysis		*p*	Multivariable analysis		*p*
	Regression coefficient	95% CI		Regression coefficient	95% CI	
Sex (male)	0.08	−4.24, 4.41	0.97	−0.95	−6.10, 4.20	0.64
Age (y)	0.57	0.13, 1.00	0.016*	0.79	0.08, 1.50	0.036*
Haller index	0.47	−1.61, 2.55	0.62	−0.75	−2.87, 1.36	0.38
Costal cartilage excision	−0.40	−4.62, 3.83	0.83	−2.05	−5.99, 1.90	0.22
Number of bars	1.50	−3.66, 6.66	0.52	0.50	−5.59, 6.60	0.83

*Note:* Postoperative pain severity was assessed using the numerical rating scale on postoperative day 5. Multivariable analysis included sex, age, Haller index, costal cartilage excision, and the number of bars inserted.

Abbreviation: CI, confidence interval.

* *p* < 0.05 indicates statistical significance.

Postoperative outcomes, including NRS scores, the number of epidural bolus administrations, and the frequency of additional analgesic use, were further analyzed based on age and sex. Surgical outcomes and group comparisons are summarized in Table 3. No significant differences were observed in postoperative complication rates or lengths of hospital stay between age or sex groups. Regarding postoperative complications, three patients (7.5%) underwent bronchoscopy under general anesthesia for atelectasis, and one patient required chest drain insertion for pneumothorax.

**TABLE 3:** Postoperative outcomes, stratified by age and sex.

	Overall	Younger	Older	*p*		Male	*p*
	(*n* = 40)	(*n* = 25)	(*n* = 15)		(*n* = 13)	(*n* = 27)	
Postoperative complication	4 (10%)	4 (16%)	0 (0%)	0.28	2 (15%)	2 (7%)	0.58
Time until eating at least half of the meal (days)	2 (1–3)	2 (1–3)	1 (1–3)	0.67	2 (1–3)	2 (1–3)	0.75
Time until resuming walking (days)	3 (3–4)	3 (3–4)	3 (3–5)	0.47	3 (3–4)	3 (3–5)	0.75
Length of hospital stay (days)	8 (7–9)	8 (7–9)	8 (7–9.5)	0.61	7 (6–10)	8 (7–9)	0.58
Time until epidural anesthesia catheter removal (days)	4 (3–5)	4 (3–5)	5 (3.5–5)	0.43	4 (3–5)	4 (3–5)	0.70
Epidural analgesia continued on POD 5	18 (45%)	10 (40%)	8 (53%)	0.52	5 (38%)	13 (48%)	0.74
Epidural bolus administration on POD 5	8 (20%)	5 (20%)	3 (20%)	1.00	4 (31%)	4 (15%)	0.40
Number of NSAID administrations	4.5 (2–14.3)	3 (1–10)	10 (3–18.5)	0.13	6 (2–11)	4 (2–14.5)	0.92
Number of acetaminophen administrations	23.5 (14.8–30.3)	22 (14–30)	27 (20.5–30.5)	0.39	25 (16–31)	22 (14.5–30)	0.57

*Note:* Continuous variables are presented as the median (interquartile range [IQR]), and categorical variables as *n* (%).

Regarding sex‐related differences in pain outcomes, no significant differences in NRS scores on POD 1 or POD 5, the number of epidural bolus administrations, or the frequency of additional analgesic use were observed between female and male patients (Figure 3). Similarly, no sex‐related differences in NRS scores on POD 1 or POD 5 were observed within either the younger or older age groups (Figure 3). In contrast, older patients reported significantly higher NRS scores compared to younger patients on both POD 1 (*p* = 0.022) and POD 5 (*p* = 0.018) (Figure 4). The median NRS scores in the older and younger groups were 7.5 (IQR, 6.5–8.0) and 4.0 (IQR, 3.0–4.5), respectively, on POD 1, and 7.0 (IQR, 6.3–7.3) and 2.0 (IQR, 1.3–2.8), respectively, on POD 5. Despite these variations in pain severity, no significant differences in the number of epidural bolus administrations or the frequency of additional analgesic use were found between the two age groups (Figure 4). Although no significant differences were observed in the frequency of either NSAID (*p* = 0.13) or acetaminophen (*p* = 0.39) administration between the age groups, NSAIDs tended to be used more frequently in the older group (Table 3). In the sex‐based analysis, the patterns of analgesic use throughout the postoperative course were nearly identical between female and male patients (Figure 1). Conversely, in the age‐based analysis, these patterns began to diverge after POD 3, indicating a trend toward consistently higher analgesic use in the older group; however, this difference was not statistically significant (Figure 2).

**FIGURE 3:**
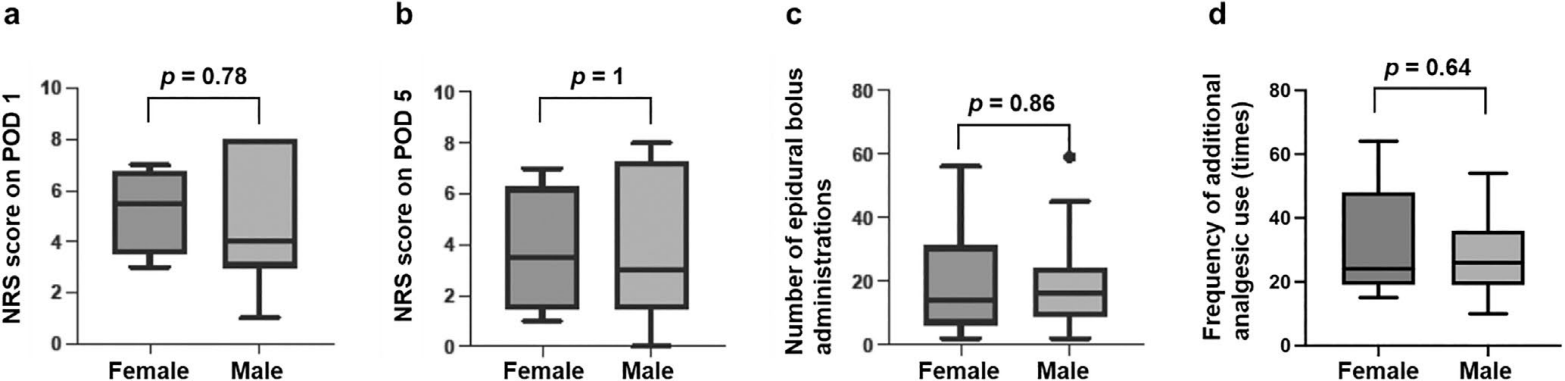
NRS scores on (a) POD 1 and (b) POD 5, (c) the number of epidural bolus administrations, and (d) the frequency of additional analgesic use, stratified by sex. No significant differences were observed. Data are presented as median (interquartile range).

**FIGURE 4:**
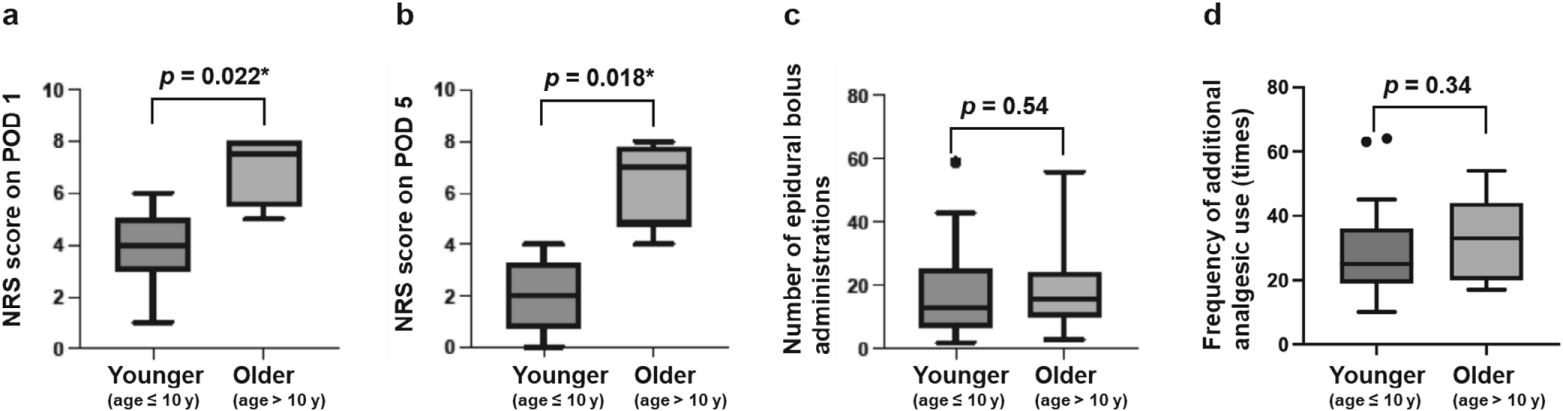
NRS scores on (a) POD 1 and (b) POD 5, (c) the number of epidural bolus administrations, and (d) the frequency of additional analgesic use, stratified by age. Older patients (age > 10 years) had significantly higher NRS scores than younger patients (age ≤ 10 years); no other significant differences were observed. *p < 0.05 indicates statistical significance. Data are presented as median (interquartile range).

## 4 Discussion

The Nuss procedure involves the forcible correction of a deformed thorax and often results in significant postoperative pain [3]. Thus, a thorough evaluation of postoperative pain and the identification of its risk factors are crucial for optimizing pain management strategies. Previous studies have investigated factors associated with postoperative analgesic requirements following the Nuss procedure in preschool children [12]. However, this study identified age as the sole predictor of severe postoperative pain in pediatric patients undergoing the Nuss procedure across the peri‐pubertal period. Pediatric patients aged > 10 years experienced more intense postoperative pain, whereas no significant sex‐related differences in pain severity were found. Additionally, neither age nor sex appeared to be associated with postoperative outcomes. Our study extends previous findings by demonstrating that age is associated with postoperative pain severity across the peri‐pubertal period.

In this study, the Nuss procedure was performed for pectus excavatum in pediatric patients aged between 6 and 19 years, a period that coincides with adolescent growth spurts. During this developmental phase, the sternum gradually undergoes ossification and increases in volume [13], and rib cartilage also begins to ossify [14]. These physiologic changes increase the force required to elevate the sternum, potentially leading to more severe postoperative pain in older patients [15]. Supporting this mechanism, a prior study that assessed the force required to elevate the sternum during the modified Ravitch repair found that this force increased with age [16]. Specifically, adolescent patients aged 11–18 years required approximately double the force needed in younger patients to achieve adequate sternal elevation [16]. These findings offer a plausible biomechanical explanation for our observation of older patients reporting more severe postoperative pain following the Nuss procedure.

In contrast to previous studies that have investigated postoperative pain in pediatric surgical populations [7, 8], we did not find a significant association between sex and the severity of postoperative pain. However, among adult patients undergoing the Nuss procedure, female patients have been reported to experience more intense postoperative pain than their male counterparts [17]. Although the mechanisms underlying sex differences in pain perception are not fully understood, several studies suggest that female patients frequently report higher levels of anxiety and postoperative pain [7, 8, 18]. Anxiety has been identified as a significant factor influencing postoperative pain perception, particularly in female patients. The discrepancies between our findings and those of previous reports may also reflect variations in ethnic or cultural backgrounds, as most prior studies were conducted in Western populations. For example, one study indicated that Asian children had lower odds of receiving analgesic medications and experiencing severe pain compared to White non‐Hispanic children. Thus, cultural differences in pain perception and reporting by patients or caregivers may partially account for the inconsistencies observed between prior studies and our results [19, 20].

Our findings reveal that a substantial proportion of pediatric patients, particularly older individuals, experience severe postoperative pain following the Nuss procedure. Although the frequency of rescue analgesic administration was comparable between the two age groups, older patients tended to receive NSAIDs more frequently than younger patients, although the difference was not statistically significant. This nonsignificant trend may reflect a greater need for escalation of analgesic treatment in older patients despite a similar frequency of rescue analgesic administration. Many older patients continued to experience severe postoperative pain despite standardized thoracic epidural analgesia and multimodal postoperative pain management. These findings suggest that older children should be recognized as a high‐risk group for postoperative pain. Accordingly, postoperative pain management protocols should take patient age into consideration, with closer postoperative pain monitoring and earlier optimization of multimodal analgesia in older children.

Standard postoperative pain management typically involves a combination of thoracic epidural analgesia and intravenous analgesic medications [21]. Despite the consistent application of this strategy within our cohort, many patients continued to report severe postoperative pain for up to five days post‐surgery. Previous studies have similarly indicated that approximately half of patients still require analgesic medications at the time of hospital discharge (POD 4) due to persistent pain [5, 22]. Inadequately managed postoperative pain can significantly diminish the quality of life in pediatric patients [6, 23], contribute to respiratory complications such as atelectasis during the early postoperative period [4, 5], and increase the risk of chronic postoperative pain persisting beyond one month [24]. The incorporation of novel analgesic approaches, such as intercostal nerve cryoablation during the Nuss procedure, may improve postoperative pain control [25]. Intercostal nerve cryoablation has been increasingly adopted internationally; however, its use remains limited in Japan, partly because of insurance and device‐access constraints [26]. In regions where cryoablation is not widely available, such as Japan, optimizing conventional multimodal analgesia remains essential, particularly given the unresolved concerns regarding its long‐term safety [27].

Although this study did not evaluate such interventions, previous studies have demonstrated a correlation between preoperative anxiety and postoperative pain [7, 8, 17]. In addition, younger children may express pain differently from older children or adults because of developmental and behavioral differences, and psychological immaturity may influence postoperative pain perception and reporting. Preoperative interventions, including electronic educational programs or psychological assessments, may alleviate anxiety in pediatric patients and their caregivers, thereby offering an additional strategy to improve postoperative pain management [28]. Overall, our findings suggest that age may serve as a practical marker for identifying pediatric patients at increased risk of severe postoperative pain, thereby facilitating risk‐stratified postoperative pain management.

Although our study included pediatric patients, all participants were school‐aged children (≥ 6 years), for whom the NRS is considered a reliable tool for pain assessment [29, 30]. Therefore, we believe that postoperative pain was appropriately evaluated in this cohort. Nevertheless, several limitations of this study warrant acknowledgment. First, its retrospective design inherently restricted the collection of certain data, such as preoperative anxiety levels. Second, minor variability in postoperative pain management practices among clinicians was unavoidable due to the study's retrospective nature, potentially limiting the generalizability of our findings. Third, this investigation was conducted at a single center with a relatively small sample size. Therefore, larger, multicenter prospective studies are needed to further elucidate predictors of postoperative pain and to establish optimized, evidence‐based pain management strategies for pediatric patients undergoing the Nuss procedure.

## 5 Conclusion

In conclusion, older pediatric patients reported more severe postoperative pain following the Nuss procedure compared to younger patients, with no significant sex‐related differences in pain severity observed. These findings suggest that age may be a practical marker for identifying pediatric patients at increased risk of severe postoperative pain and should be considered when planning postoperative pain management following the Nuss procedure.

## Author Contributions

**Sayaka Matsumoto:** conceptualization, methodology, investigation, writing – original draft. **Koichi Deguchi:** conceptualization, writing – review and editing, supervision. **Naoko Uga**, **Tasuku Kodama**, **Kengo Nakahata**, **Motonari Nomura**, **Takehisa Ueno**, and **Miho Watanabe:** writing – review and editing. **Hiroomi Okuyama:** writing – review and editing, project administration.

## Funding

The authors have nothing to report.

## Ethics Statement

This study was approved by the Ethical Review Board of Osaka University Hospital (approval number: 24194) and was conducted in accordance with the Declaration of Helsinki. Information regarding the study was disclosed to patients, with the opportunity to opt out of participation.

## Conflicts of Interest

The authors declare no conflicts of interest.

## Data Availability

The data that support the findings of this study are available on request from the corresponding author. The data are not publicly available due to privacy or ethical restrictions.
